# SNP markers retrieval for a non-model species: a practical approach

**DOI:** 10.1186/1756-0500-5-79

**Published:** 2012-01-29

**Authors:** Arwa Shahin, Thomas van Gurp, Sander A Peters, Richard GF Visser, Jaap M van Tuyl, Paul Arens

**Affiliations:** 1 Wageningen University and Research Centre, Plant Breeding, P.O. Box 16, 6700 AJ Wageningen, The Netherlands; 2 Wageningen University and Research Centre, Bioscience, P.O. Box 16, 6700 AJ Wageningen, The Netherlands; 3 Netherlands Institute of Ecology (NIOO-KNAW), Department of Terrestrial Ecology, P.O. Box 50, 6700 AB Wageningen, The Netherlands

## Abstract

**Background:**

SNP (Single Nucleotide Polymorphism) markers are rapidly becoming the markers of choice for applications in breeding because of next generation sequencing technology developments. For SNP development by NGS technologies, correct assembly of the huge amounts of sequence data generated is essential. Little is known about assembler's performance, especially when dealing with highly heterogeneous species that show a high genome complexity and what the possible consequences are of differences in assemblies on SNP retrieval. This study tested two assemblers (CAP3 and CLC) on 454 data from four lily genotypes and compared results with respect to SNP retrieval.

**Results:**

CAP3 assembly resulted in higher numbers of contigs, lower numbers of reads per contig, and shorter average read lengths compared to CLC. Blast comparisons showed that CAP3 contigs were highly redundant. Contrastingly, CLC in rare cases combined paralogs in one contig. Redundant and chimeric contigs may lead to erroneous SNPs. Filtering for redundancy can be done by blasting selected SNP markers to the contigs and discarding all the SNP markers that show more than one blast hit. Results on chimeric contigs showed that only four out of 2,421 SNP markers were selected from chimeric contigs.

**Conclusion:**

In practice, CLC performs better in assembling highly heterogeneous genome sequences compared to CAP3, and consequently SNP retrieval is more efficient. Additionally a simple flow scheme is suggested for SNP marker retrieval that can be valid for all non-model species.

## Background

In the last few years, the development of next-generation sequencing technologies that have the capacity to generate millions of short reads in a single run, has led to a revolution in sequencing applications. The NGS technologies not only boosted re-sequencing and allele mining studies in model species, but are also very useful for the development of SNP markers in species with no or hardly any genetic resources.

SNP development using NGS technologies essentially has become cheaper and faster but also generated requirements like the need for genome complexity reduction, assembly of sequences, and SNP identification in high throughput. The latter two steps are still considered challenging. Currently many different assemblers are available, but few studies discussed the performance of different assemblers in relation to assembly quality and the influence of genome complexity and heterogeneity on the quality of the assembly. Assembly quality is generally assessed by: the lengths of the contigs (mean, minimum and maximum lengths, or N50 according to the assembler), and the accuracy or correctness of the assembly (how well the contigs can be mapped to the reference genome) [1]. Two different assemblers (Newbler and MIRA) were compared on an insect sequence dataset using public Sanger EST data and 454 transcriptome data [2]. Another study compared six assemblers (CAP3, MIRA, Newbler 2.3, Newbler 2.5, SeqMan, and CLC) in reference to the number and length of contigs, speed of assembly and assembly redundancy in *de novo *assembly of a nematode [3]. The quality of the contigs was checked by aligning the contigs to four reference sequence sets (ESTs, proteome, gene families, and protein data from databases). Similarly, the performance of six aligners (BLAT, SSAHA2, Bowtie, SeqMap, MAQ, and CLC) were compared using *in silico *generated transcripts from four model organisms (human, *Arabidopsis*, *Drosophila*, and yeast) that were mapped to the transcriptome or the complete genome from sequence databases [4]. Results showed that with increasing sequence read length mapping was more accurate, while with increasing genome heterozygosity more reads were incorrectly mapped. Recently, a comparison in which eight short reads assemblers were evaluated against two types of simulated short reads datasets (allowing 0.1% error rate) derived from four different genomes (nematode, yeast, bacteria, and virus), was published [5]. The assemblers' performance information about computational time, memory cost, assembly accuracy and completeness and size distribution of assembled contigs where studied (by mapping to reference genomes) [5]. All these studies used relatively small sized genomes, and often inbred organisms and studied assembly accuracy in general parameters and by mapping to reference genomes or Sanger sequencing data [2-5]. Additionally these studies showed that there is currently no commonly accepted and standardized method for performance evaluation of assemblers, none of these studies checked the assembly quality concerning SNP markers retrieval, and no clear guidance for assembler selection was defined. Because we are involved in ornamental breeding where, in general, crops are outcrossing and highly heterogeneous without reference sequences, our goal was to study the effects of two different assemblers on assembly performance and SNP retrieval in heterogeneous outcrossing species by using our model crop lily as an example. Running such a study, conformation of assembly quality by mapping to a reference genome would be optimal. However, species with reference genomes do not represent the same level of heterogeneity and genome complexity as is found in most outbreeding non-model species. In our study we analyzed a highly divergent sequence dataset of the non-model species lily that allows us to investigate a real case study and develop a flow scheme that can be followed in SNP marker development studies for similar non model species.

When working with *Lilium*, which has an assumed high level of diversity, a large genome size of 36 Gb with an accompanying high genome complexity and a lack of genetic resources, assemble is an important step in SNP retrieval. Since clear criteria on choosing an assembler are lacking, in our study we focused on two widely used assemblers (CAP3 and CLC) which represent the two different approaches which are used in assemblers. CAP3 is selected since it uses the overlap algorithm for assembly and was successfully used to assemble EST genebank data in heterozygous species such as *Zea mays *[6] and potato [7,8]. Recently it was used to assembly apricot (*Prunus armeniaca L.)*, castor bean, mulberry (*Morus sp*.), Pigeonpea (*Cajanus cajan *L.), rice, and grape [9-15]. Furthermore, CAP3 is implemented in the QualitySNP pipeline [7] which is a pipeline to identify SNPs and was used in SNP mining studies [8,16]. CLC assembler is selected since it uses the de Bruijn algorithm, it was used in several comparison studies and showed to produce a good quality assembly [3,4]. It is a user friendly assembler since it is not a command line programming software and it has a complete package (cleaning, trimming, clonality removal, SNP and InDels counting, and assembly, in addition to a very advanced visualization technique of the assemblies) which make it a very appealing software to be used. Moreover, CLC assembler supports both short read and long read assembly, and also supports *de novo *assembly of paired end data. Also, CLC was used because it was indicated to perform better in mapping of artificial datasets with increased heterogeneity [4]. Additionally, recent papers on the performance of assemblers used both assemblers [3,17], which indicates the importance and usability of both assemblers.

In this study CAP3 and CLC were used for *de novo *assembly of 454-transcriptome reads derived from *Lilium*. The goals of this study were: 1) comparing the performance of CAP3 and CLC by running *de novo *assembly, 2) show the influence of the assembler on the reliable detection of alleles and SNPs, and 3) suggesting a simple flow scheme to generate reliable SNP markers out of such heterozygous species.

## Results and discussion

### Pre-processing step

In this study, we generated a large number of genes from the genus *Lilium*. In total, 1,282,735 reads with an average length of 340 bp were derived using 454 pyrosequencing. The lowest number of reads was obtained from 'Connecticut King' (139,480) reads and the highest from the Trumpet genotype (442,476 reads). From 'White Fox' and 'Star Gazer' 326,539 and 374,240 reads were obtained respectively. This difference in the number of reads might be related to the quality of RNA that was used for each genotype and variations in the initial amount of cDNA that was used of each sample for sequencing.

Cleaning the data showed that 85,719 reads (6.7%) were discarded either because of poor quality, being too short (less than 100 bp), being too long (over 800 bp), or missing the barcode sequence. Around 1,191,938 reads with an average length of 283 bp (after trimming) were kept for further analysis.

Next, all the duplicated reads were removed. The presence of duplicated reads affects the reliability of a SNP call. In sequence data analyses for SNP retrieval, reads are assumed to be from independently derived DNA fragments. Any polymorphism event present independently twice, will be considered as reliable whereas polymorphisms found independently only once could also be due to possible mistakes in cDNA synthesis and PCR steps. Duplicated reads with PCR mistakes still present in sequencing data could result in the selection of these mistakes as SNP and therefore should be avoided. The number of initial transcripts and the effects of differential amplification in the preparation of the sequencing libraries determine the final library output quality (goal is the presence of a variety of transcripts as wide as possible), and thereby affects the percentage of duplicated reads. The more diverse a library is the less duplicated reads. All the clonal reads were excluded (412,826 reads, 35%) and only the longest of the clonal reads were retained leaving a total of 779,112 reads (220,716,355 bp) for the assembly step. Similar results on clonal reads were detected in previous studies in which 11% to 35% of the sequences were reported as potential artificial replicates (e.g. [18]). Gomez-Alvarez [18] suggested that this phenomenon could be explained by the binding of amplified DNA fragments generated in the emulsion PCR step of the 454 pyrosequencing to empty beads. However, clonality of reads is not limited to a specific mechanism since it was recorded in GS20, GS-FLX, and GS-FLX Titanium systems [18] as well as in Illumina's Solexa [19] which indicates the possibility of another explanation. The relative high clonality found with different sequencing technologies could be related to the cDNA library preparation in which often PCR steps are used to generate sufficient quantities of cDNA for sequencing [19]. In particular, the second PCR after the normalization step (using the primers adapters of the A and B adapters) may increase the number of duplicated reads. In our case, we could detect duplicated reads since no shearing of fragments was applied but instead fragments were generated by using randomized primers for cDNA synthesis, adapter primers were used in the first PCR step and size selection was obtained by gel electrophoresis. The same way of cDNA synthesis and normalization was also applied in other studies [20,21]. However, none of these checked for duplicated reads. Library construction and normalization protocols minimizing PCR steps and preventing the occurrence of duplicate reads would be preferable [19]. Nevertheless, data should always be checked for duplicated reads in order to remove them.

### Assembly and SNPs detection

#### CAP3 assembly

**Figure 1:**
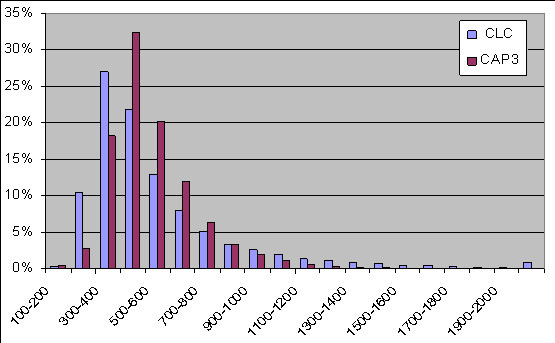
**The distribution percentage of CLC and CAP3 contigs length**. The distribution percentage of contig lengths assembled by CLC and CAP3 assemblers.

CAP3 uses an overlap-layout consensus algorithm for cluster construction and as such is suitable for SNP mining [22], although it is a relatively slow assembler. EST data were clustered by CAP3 with a stringency level of 95% similarity per 100 bp. The CAP3 alignment resulted in 576,882 reads that were assembled in 72,540 contigs (38.4 Mb) with an average of 8 reads per contig (Table 1). Around 26% (202,230) of the reads were singletons. The average length of the contigs was 530 bp, 274 contigs (0.38%) were less than 200 bp in length. Around 2.5% (1780) of the contigs were longer than 1 Kb, 4 contigs were longer than 2 Kb of which the longest contig was 2,800 bp (Figure 1). A total of 10,461 reliable SNP markers were identified by QualitySNP [7].

**Table 1:** Comparison between CAP3 and CLC assembly results

	CAP3	CLC
**Nr contigs**	72,540	55,433

**Average contig length**	530 bp	555 bp

**Assembled**	576, 882	646,424

**Nr singletons**	202,230	132,688

**Average reads/contig**	8	11.66

**Nr of SNP markers**	10,461	2,421

#### CLC assembly

CLC uses the de Bruijn algorithm which is used in several assembler software packages such as Velvet [23], Oases http://www.ebi.ac.uk/~zerbino/oases/[24], ABySS [http://www.bcgsc.ca/platform/bioinfo/software/abyss][25], and SOAPdenovo [http://soap.genomics.org.cn/soapdenovo.html][26]. Similar to CAP3, a cutoff threshold of 95% was used which resulted in the assembly of 646,424 reads in 55,433 contigs (30.8 Mb) with an average of 12 reads per contig (Table 1). Around 17% (132,688) of the reads were left out as singletons. The average length of the contigs was 555 bp, 177 contigs (0.32%) were less than 200 bp in length. Around 8.5% (4709) of the contigs were longer than 1 Kb, 485 contigs exceeded 2 Kb of which the longest contig was 9,420 bp (Figure 1). A total of 2,421 SNP markers were identified by QualitySNP as reliable markers.

A reasonable percentage of the *Lilium *transcriptome was covered as could be estimated from the transcriptome size of the monocot model species rice, which has 41,000 genes with average gene length of 2,000 bp [27]. Assuming that lily has a comparable transcriptome size, the CAP3 contigs cover around 47% of the *Lilium *transcriptome while the CLC contigs cover 38%, regardless of the singletons that could be added to the total coverage.

Notable differences between the assembler's performance were recorded in this study. Similarly, differences in assembler's performance were also found in another study [28]. The performance of different assemblers (Velvet, Oases and SeqMan NGen) were compared on a non-model species (snail) and showed that the assembly is strongly depend upon the assembler [28]. In this study, CLC assembled more reads compared to CAP3 and also generated longer contigs with a higher average read coverage. However, CAP3 contigs generated more SNP markers and appeared to have a higher coverage in total sequence length. Both assemblers in addition to several other aligners were compared considering the number and mean length of the contigs, the assembled reads, and the assembly redundancy [3]. In contrast to our results, CAP3 and CLC performed comparable in their study. To our knowledge, there are no studies published in which the assembler's performance has been evaluated with respect to SNP retrieval. SNP markers will segregate nicely in mapping studies if the SNP is true (reliable) and the marker is unique throughout the genome (high quality). The first step to generate reliable and high quality SNP markers is building contig in which alleles are joined and paralogs are preferably separated.

In order to choose the best assembler with respect to the identification of high quality reliable SNP markers for genetic mapping, we performed several tests to compare the performance of the assemblers.

### Comparison between the CAP3 and CLC assemblies

#### Assembly redundancy

**Figure 2:**
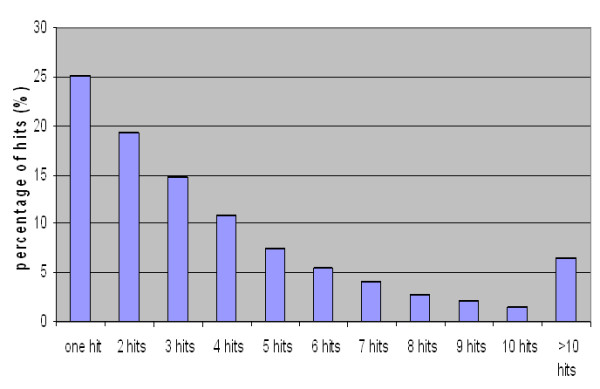
**CAP3-contigs blast vs. CLC contigs**. This graph present the number of hits resulted by blasting the CAP3 contigs vs. CLC contigs. Around 25% of the CAP3 contigs had one hit and all the rest (75%) have more than one blasting hit with CLC contigs.

Redundancy is a main parameter in which the quality of assemblies can be evaluated. Redundancy occurs when different contigs are likely to originate from the same locus as defined by the degree of similarity [2]. This is related to high numbers of differing bases which may be due to alternative splicing, multiple SNPs, InDels, and mismatches and misalignments due to homo-polymer tracts all of which show high frequencies in an outbreeding and highly heterozygote species such as in our case. The best assembler will assemble the largest number of unique sequences regardless of the number of contigs. A high redundancy of contigs is an indicator of poor assembly [3]. A pair wise comparison was performed by blasting the contigs of CAP3 vs. CLC-contigs with a threshold of E-20. This comparison will help to verify if the differences in contigs size between the two assemblies were related to novel sequences or to the presence of repetitive and redundant contigs [3]. Results showed that only 25% of the CLC-contigs have a unique blast hit to a single CAP3 contig (Figure 2). Similarity values of all hits exceeded E-45 except for a few cases where it ranged between E-20 and E-35 with identities above 90%.

**Figure 3:**
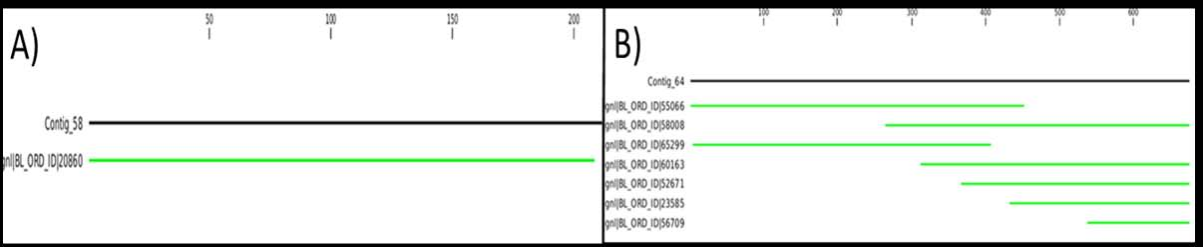
**Configuration of two examples of blasting CAP3 contigs vs. CLC contigs**. **A**) in this example one contig of CAP3 assembled with one contig CLC, **B**) seven contigs of CAP3 grouped in one contig of CLC.

Visual inspection of the blast hits showed that in some cases both assemblers have mapped the same reads and constructed identical contigs (see Figure 3a). However, in most cases several CAP3-contigs mapped to one CLC-contig (Figure 3b) which indicates a high level of redundancy in CAP3 contigs. Blast results from blasting all CAP3 contigs among themselves confirm this (results not shown). These results indicated a significant difference in assembly performance between the two assemblers. This might be explained by the fact that the two assemblers use different approaches. CAP3 uses OLC (Overlap-Layout-Consensus) in assembling the data (which is also used in MIRA, Newbler, and SeqMan), while CLC uses de Bruijn graph path finding (which also are used by Oases, Velvet and ABySS). OLC compares the overlap of whole reads at once while de Bruijn compares small stretches of base pairs (*k*-mers, 21 in our case) and combines all similar reads in one contig. This difference in algorithms may have made CLC more able to assemble reads with a high level of heterozygosity compared to CAP3 which showed to be highly discriminating. These differences between the performances of the two assemblers were not recorded in a previous study [3]. A possible explanation could be in differences in the level of heterozygosity present in the cDNA sequences between the studies. Kumar and Blaxter [3] used the cDNA of a model filarial nematode that has low levels of heterozygosity (Mark Blaxter, pers. comm.), while lily is an outcrossing species with a very heterogeneous breeding pool that has a high level of polymorphism (SNPs, InDels) and combined with mistakes introduced by 454 pyrosequencing (especially in homopolymer tracts) makes the sequence reads highly heterogeneous. The level of heterozygosity within lily cultivars is around one SNP per 50 bp (calculated based on a random set the cDNA sequences), and among the four cultivars around one SNP per 30 bp. The more polymorphisms present in sequence reads, the more divergence can be detected in the performance of assemblers. The effect of genome complexity on mapping was highlighted by Palmieri and Schlotterer [4]. This study showed that complex genomes, containing many gene families and paralogs are more difficult to be mapped to a reference genome compared to a compact genome. It was also recorded that SeqMan (OLC strategy) was not able to map reads (100 bp) that contain 9% computer generated variation due to the high divergence of the reads whereas this was feasible with CLC [4]. The differences in abilities to deal with genome complexity and heterogeneity among sequences of the assemblers indicate the importance of assembler choice.

#### Contigs blast to public sequence data base ESTs

**Figure 4:**
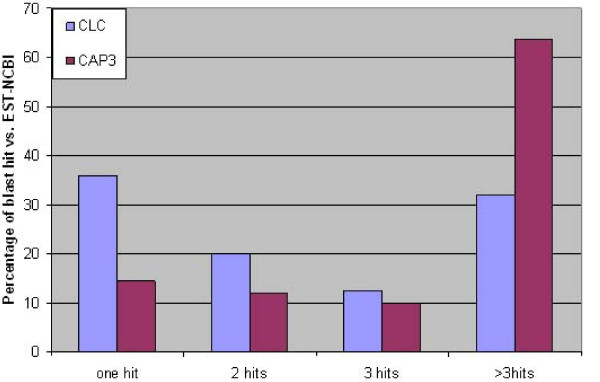
**Blast contigs against EST-NCBI**. The contigs assembled by CLC and CAP3 were blasted separately vs. EST-NCBI (BlastN, threshold 1E-20).

**Figure 5:**
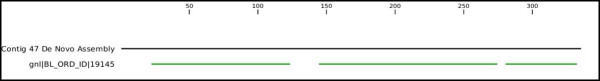
**Configuration of blasting CLC-contigs vs. EST-NCBI**. A partial matching of CLC contigs vs. EST-NCBI sequence.

In the majority of studies using NGS technologies, available data of the sequence database was used to support the assembly step since these sequences are relatively long compared to NSG sequences, and more reliable since they resulted from Sanger sequencing which is still considered the gold standard in terms of sequence reliability. For lily, the number of available EST data in the sequence database is very limited with 3,329 ESTs, clustering (using the default parameters of CLC) into 381 UniGenes. These UniGenes were used to compare the performance of the two assemblers by aligning the contig consensus sequences of each assembler with the 381 UniGenes and analyzing the results. CAP3-contigs showed a total of 251 hits, 86% of them were redundant (more than one BLAST-hit) compared to CLC that showed 260 hits of which 64% showed redundancy (Figure 4). Although these results seem comparable, there also seem to be differences here since for CLC the contigs mainly assembled adjacent to each other (Figure 5) rather than to the same sequence stretch as was often found for CAP3-contigs. This means that several short but unique contigs of the CLC and CAP3 assembly are positioned within the EST sequence. Thus, the use of EST data from the databases to assess the performance of the two assemblers is not informative if not the two former cases (overlap or adjacent alignment) can be well defined and distinguished.

#### Blasting generated SNPs *vs*. the contigs

Blast results from QualitySNP selected SNPs (with 50 bp flanking sequence on each side) vs. the contigs from the assembly they originated, provided an additional criterion for SNP markers selection. Many species have undergone genome duplications during their evolution. Assuming paralogs are assembled in different contigs it is still possible that SNP markers selected from one of these contigs will also be present in a paralogous gene assembled in another contig. Thus, is vital to check that SNP markers only map back to the contig from which they were selected. This paralog detection is important in any study aiming to generate SNP markers for which other genetic resources are missing. Selected SNP marker sequences (101 bp) of each assembler were blasted against all contigs using a threshold of E-20.

**Figure 6:**
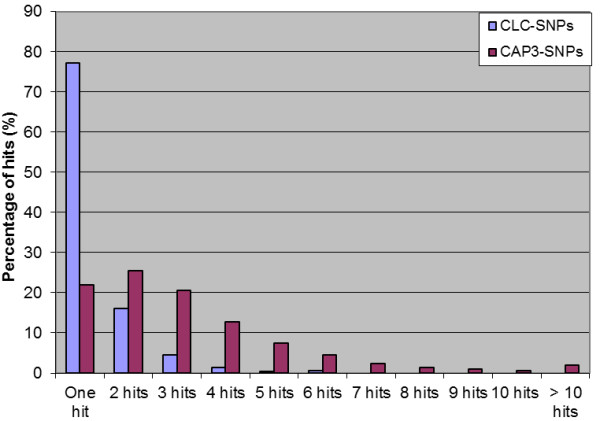
**The percentage of hits resulted from blasting SNPs vs. the contigs**. The CAP3-SNP (101 bp) were blasted vs. CAP3-contigs, and CLC-SNP (101 bp) were blasted vs. CLC-contigs, and the number of hits were recorded.

The CAP3-SNP blasting showed that only 22% of the generated 'SNP markers' (defined here as the SNP and 50 bp flanking sequence on each side) uniquely mapped to the contigs from which they were derived, 78% of the SNP markers had more than one blast hit, and 198 SNP markers had more than 10 blast hits (Figure 6). This indicated that CAP3-SNP markers were not unique due to either a high percentage of paralogs in the *Lilium *genome or due to poor assembly and thus cannot be used for mapping studies. However, around 83% of the SNP markers generated in a CAP3 assembly of 454 transcriptome pyrosequencing in the inbreeding species *Solanum lycopersicum*, in which the level of polymorphism is low, showed to be unique (Dr. AW van Heusden, Wageningen UR Plant Breeding, pers. comm.). From this, we can conclude that the performance of CAP3 is negatively correlated to the polymorphism level present in the genome studied. In the case of high heterozygosity as in the present study, CAP3 software might separate alleles of highly polymorphic loci into different contigs which means that these contigs are not unique and thus it is highly risky to use them to generate SNP markers. Redundant contigs can either be related to paralogs or they can be alleles of the same locus (among the four genotypes) that were split up into different contigs. In both cases the SNP markers should not be used for mapping purposes. In case of paralogs, SNP markers will cause problems in SNP detection. In the latter case, there is a chance that SNP markers will either give no call or will work poorly because the risk of secondary SNPs close to the SNP of interest is overlooked. So, the most trustworthy SNP markers will be the ones that were generated when all alleles of the same locus are grouped in one contig. CAP3 generated 5775 contigs (8%) that include sequences of the four cultivars, compared with 9234 contigs (17%) for CLC.

In CLC, 77% of SNP markers were unique. Only 13 SNP marker sequences had more than 5 blast hits (Figure 6). The 22% of redundancy among CLC-SNPs can be related to the presence of paralogs assembled in different contigs. In general, a number of genes in any genome are expected to be duplicated especially in case of a huge genome like that of *Lilium*. The percentage of paralogous genes differs between species. For example, in rice around 15 to 62% were expected to be duplicated genes [29]. Using a strict method of defining paralogs, the 22% of redundancy among CLC-contigs is more in line with expectations than the 78% among CAP3 contigs, especially when taking in consideration that not all paralogous genes will be expressed at the time of sampling. To check whether CLC combined paralogs in contigs, haplotype numbers were assessed. Only, 0.7% (364) of the CLC-contigs combined paralogs and contained more than the maximum expected 8 alleles (expected of 4 heterozygote diploid cultivars). The actual number of CLC-contigs with paralogs may be slightly higher but is not likely to cause high numbers of erroneous SNP markers in mapping. Thus, CLC appeared to perform reasonably well for SNP markers retrieval even with the sequence data of this highly polymorphic species. This is in correspondence with [4] where CLC was among the two best programs for *de novo *sequence assembly. In contrast, CAP3 could not handle such high levels of heterogeneity [4].

#### Examples of assembly differences between the two assemblers

**Figure 7:**
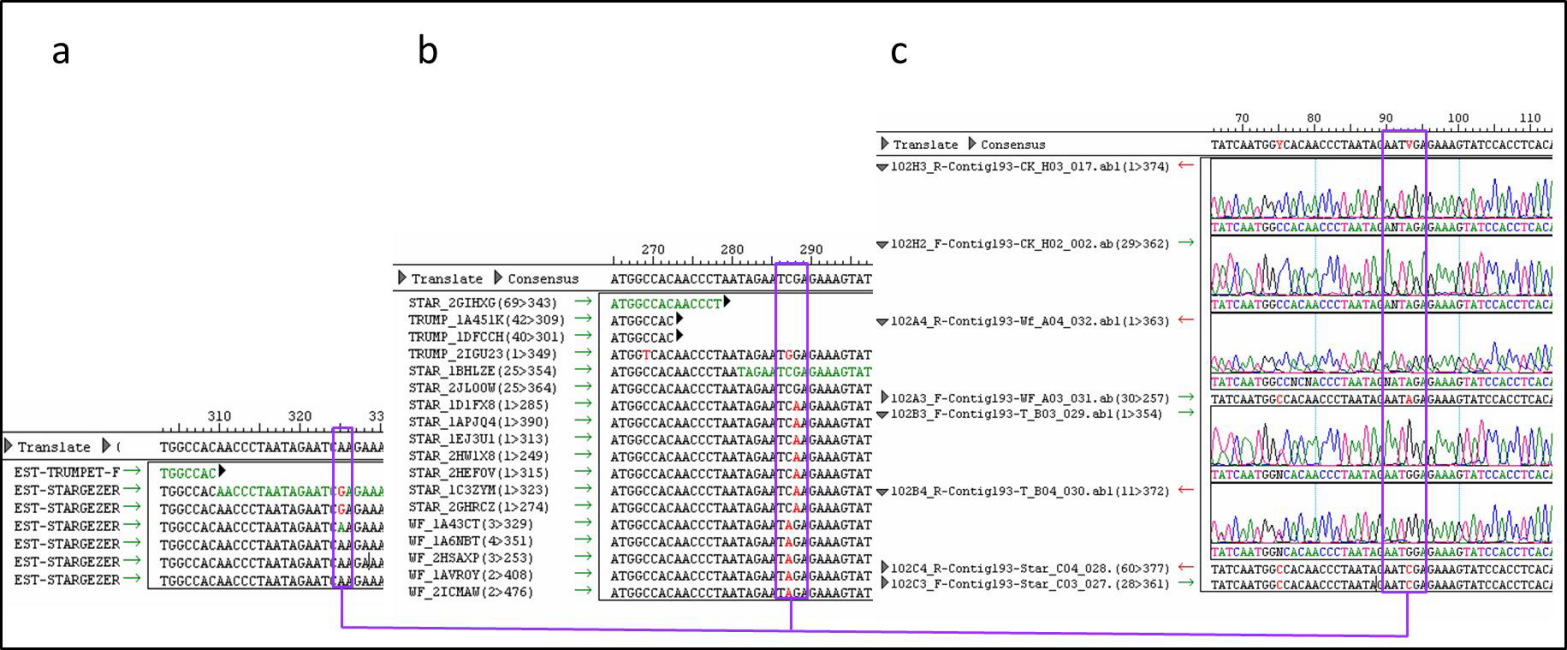
**Comparing the same 454 sequences assembled by CAP3 and CLC from one side and with Sanger sequence of the same contig assembled by SeqMan on the other side**. a) contig 193 resulted of CAP3 assembly, b) contig 23548 resulted of CLC assembly that contains all the sequences included in contig 193 of CAP3, c) Sanger sequencing for this contig of the four cultivars (Connecticut King, White Fox, Trumpet and Star Gazer). Boxes indicate positions with SNPs. Lines connect the same SNP in the different contigs.

This step was done to visualize on the individual contig level the influence of polymorphism: on the assembly, on the SNP selection processes in each assembly and on the Quality value D. The Quality D-value is the standard deviation of the normalized number of potential SNPs among haplotypes and it is calculated and used to assess the probability that a cluster contains paralogs [7]. Randomly we selected two contigs with SNP markers. The first is a contig of CAP3 that showed a SNP marker (Contig 193) with a low Quality value (D value = 0) that indicates a high quality/reliability for this SNP. High D-values indicate a high probability that a cluster contains paralogs as well as allelic sequences (Tang et al. 2008). The contig consensus of CAP3 contig 193 was blasted against the CLC-contigs and the matching contig 23548 (D = 0.59, haplotypes = 4) was examined. Contig 23548 has no indication of a possible SNP marker. Based on the consensus sequence, primers were developed and used to amplify the putative SNP region in the CAP3 contig 193 in the four genotypes and the obtained fragments were used in Sanger sequencing to re-sequence the putative SNP region. Sanger sequences were assembled by SeqMan (Lasergene, version 8) and then compared to contig 193 from CAP3 and contig 23548 from CLC. CAP3 contig 193 contained six reads of 'Star Gazer' with an intra SNP marker (Figure 7a). In CLC, the same six reads together with three reads of 'Trumpet', five reads of 'White Fox' and four more reads of 'Star Gazer' formed contig 23548 (Figure 7b). Sanger sequences (Figure 7c) of the four genotypes confirmed that there is no 454 sequence's mistake in this locus. The A/G SNP found within 'Star Gazer' is a reliable SNP which was also shown by CLC and confirmed by Sanger (see Figure 7). However, the SNP was not selected as a candidate SNP marker in the CLC assembly by QualitySNP since very close SNPs (within 50 bp) were detected in the other genotypes and consequently this would not produce a general applicable SNP maker for Illumina Golden Gate (Figure 7b, c). This example showed clearly that the CLC assembler combined reads which were separated into two contigs by CAP3 (Contig 193 and Contig 338). This indicated that CAP3 (OLC algorithm) treats alleles and homologous sequences of the same locus as paralogs belonging to other contigs or leaving them as singletons, above certain levels of polymorphism. This might explain the difference in contig and singleton number between the two assemblers. It also explains why although each contig of CAP3 contains lower levels of polymorphisms in total, the assembly results in the identification of more candidate SNP markers compared with CLC that contained more reads per contig. This counter-intuitive situation is due to the higher numbers of flanking SNPs that are found in CLC-contigs and that limits the number of candidate SNPs that can be used as SNP marker in genotyping.

**Figure 8:**
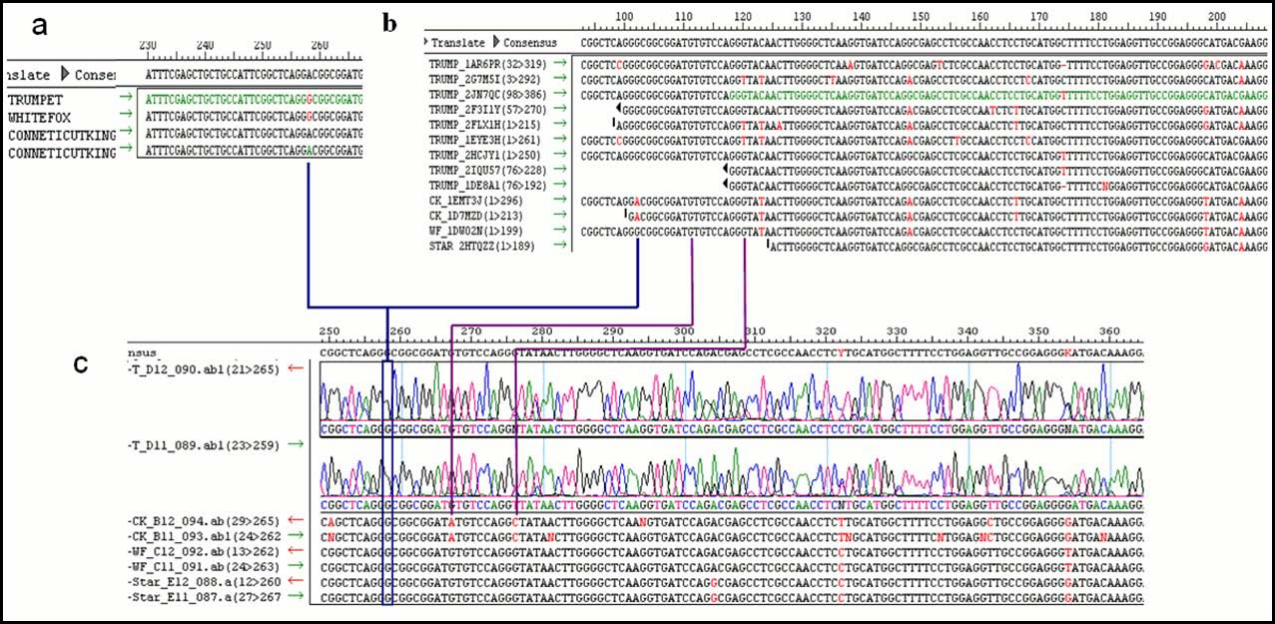
**Comparing the same 454 sequences assembled by CAP3 and CLC from one side and with Sanger sequence of the same contig assembled by SeqMan on the other side**. a) contig 63 resulted of CAP3 assembly, b) contig 12221 resulted of CLC assembly that includes all the sequences of contig 63 resulted of CAP3, c) Sanger sequences generated for this contig of the four cultivars (Connecticut King, White Fox, Trumpet and Star Gazer). Lines connect the same SNP in the different contigs.

In the second example, contig 63 of CAP3 containing four reads; two of 'Connecticut King', one 'Trumpet' and one 'White Fox' indicated a reliable inter (G/A) SNP marker with D = 0.2 (Figure 8a). The same reads grouped together with another nine reads (one of 'Star Gazer' and eight more of 'Trumpet') in the CLC assembly (Contig 12221, D = 0.6, haplotype = 5). No SNP marker was selected out of contig 12221 due to presence of other close by SNPs. Surprisingly, we could not verify the SNP in the Sanger sequences generated for all 4 genotypes using primers designed on this contig (Figure 8c). Sanger sequences indicated nucleotide (G) in all four cultivars. Another unexpected result is that the 'Connecticut King' sequences of Sanger did not match in several places with the 454 sequences (Figure 8b and 8c). Moreover, CLC clearly grouped paralogs in the contig since 'Trumpet' reads show more than two alleles (Figure 8b). By blasting the CLC contig 12221 vs. CAP3 contigs two hits (Contig 63 and Contig 66474) were found providing evidence that this SNP of CAP3 could not be used since it did not show a unique match to the contig from which it was selected.

**Figure 9:**
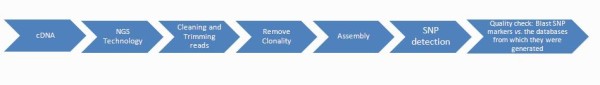
**A scheme showing the main steps proposed to generate reliable SNP markers**.

This example showed that since CLC assembly adds more reads to a contig compared to CAP3, the risk of grouping paralogs into one chimeric contig might be higher. This also indicates the importance of filtering for haplotype number per cultivar and low D-values before selecting the SNP. A maximum of eight haplotypes can be expected in the assembly of four diploid heterozygous cultivars. Out of the 2,421 SNP markers selected by CLC, 4 contigs have haplotype numbers higher than 8. Number of haplotypes in contigs gives a clue on the frequency with which paralogs are incorporated into single contigs and QualitySNP uses it in SNP identification. In an ideal situation having the full genome sequence, all SNP flanking regions can be blasted to the genome and thus SNP markers can be identified for which paralogs are present. Unfortunately, for many species in which researchers would like to use the advantages of NGS technologies to develop SNP markers, whole genome sequences will not be readily available and blasting of SNP 101 bp flanking regions vs. contigs is the best alternative. To sum up, the main steps which are needed to generate SNP markers of a non-model species are summarized in Figure 9.

## Conclusion

SNP markers are becoming the markers of choice in genetic studies and as such for many species researchers are likely to start up SNP retrieval from NGS data. Our results clearly showed that sequence assembly and consequently the SNP markers retrieval are affected significantly by the assembler. In our study, we tested two widely used assemblers that use different algorithms. Procedures followed can be used in any species that has little genetic resources to view assembly quality. Importantly, blasting the selected SNP markers vs. the contigs from where they generated from (in case of missing the support information from the databases) or against the whole genome, if available, is very essential to avoid false positive SNPs. Results obtained with *Lilium *cDNAs are likely also valid in other highly heterogeneous species. There seems to be a strong correlation between the level of heterozygosity in the studied species and the performance of the assemblers.

Overall, we believe that for inbreeding species both assemblers can be used, while in an outbreeding and highly heterozygote species CLC is preferred.

## Methods

### Plant materials

Four lily genotypes that represent the four main hybrid groups of the genus *Lilium *were used for sequencing: cv 'Star Gazer' (Oriental), breeding line 'Trumpet 061099' (Trumpet), cv 'White Fox' (*Longiflorum*), and cv 'Connecticut King' (Asiatic). Young leaves (500 mg) were collected and kept at -80°C upon RNA isolation.

### RNA isolation and cDNA library preparation

Using the Trizol protocol (Invitrogen.Carlsbad, CA, USA), the RNA of the four genotypes was isolated and subsequently purified using the RNeasy MinElute kit (Qiagen, Hilden, Germany).

RNA library processing i.e. cDNA synthesis, normalization of the cDNA and adaptor ligation for GS FLX Titanium sequencing, was performed by Vertis Biotechnologie AG (Freising, Germany). In short, 45 ug of total RNA of each of the four samples was treated with DNase and then primed with 6 nucleotide random primers for first strand cDNA synthesis. Next, 454 adapters A and B with an unique 6 nucleotides barcode for each cultivar were ligated to the 5' and 3' ends of the cDNAs. These cDNAs were subjected to two steps of PCR: one before the normalization step (around 18 cycles) and one after it (around 8 cycles) using a proof reading enzyme. Normalization was carried out by one cycle of denaturation and re-association of the cDNAs and subsequent column purification. For Titanium sequencing the cDNAs in the size range of 500-600 bp were eluted from preparative agarose gels.

### 454 sequencing procedures

The four cDNA libraries were mixed in equal concentrations and sequenced on a Life Sciences GS-FLX Titanium according to standard procedures (454 Life Sciences) at Wageningen UR Greenomics (Wageningen, the Netherlands).

### Data availability

Raw sequence data are available at ENA-SRA (European Nucleotide Archive-Sequence Read Archive) with the accession number ERP001106.

### Assembly

Raw unprocessed sequences were cleaned before assembly using both the reads and the accompanying sequence quality information (SFF files). Trimming was done by removing: 5' and 3' adapters sequences, low quality bases (limit 0.05), ambiguous nucleotides (maximum 2 nucleotides allowed), terminal nucleotides (one nucleotide from the 5' end and 15 nucleotides from the 3' end), and removal of all reads that have less than 100 and more than 800 nucleotides.

Next, all the duplicated reads, i.e. reads that have the same first 6 nucleotides and exactly the same sequence (>98% similarity), were excluded (clonality) using CD-HIT [30]. After trimming and removing clonality, all the reads were submitted to the standard CAP3 [31] using the default parameters (threshold identity cutoff 95% over 100 bp) and CLC Genomics Workbench software (CLC bio, Denmark, http://www.clcbio.com/). The *de novo *assembly using CLC was done using the following parameters: conflict resolution (vote), similarity 95% 100 bp over read length and alignment mode (global, do not allow InDels). Through this study few terms will be used frequently such as:

• Assembler's performance: refer to the number of contigs with average contig's length, the number of singletons, and assembly redundancy.

• Assembly redundancy: when the assembler tend to separate sequence related to the same locus over different contigs.

### SNP detection

All the contigs resulting from CAP3 and CLC were submitted to an updated version of QualitySNP [7] to detect reliable single nucleotide variants within each genotype (between the alleles in one genotype, intra SNPs) and between the four genotypes (between the alleles of the four genotypes, inter SNP). SNPs were chosen using the QualitySNP program based on the following criteria: high quality sequence, not within or adjacent to a homopolymeric tract, at least 2 reads of each allele, 50 bp of flanking sequence on each side free of other SNPs and InDels (criteria needed by Illumina Golden Gate platform for SNPs genotyping). Any SNP fitting these criteria is considered and referred to as 'reliable SNP marker', reliable SNP markers are referred as 'high quality' if they are uniquely present in the genome. For the latter, the SNP with 50 bp sequence on either side is compared against all contigs of the same assembler using BLASTN with Expectation value 1E -20. Only SNPs mapped uniquely to the contig from which they were selected (i.e. high quality SNPs) will be retained for marker analysis.

## Competing interests

The authors declare that they have no competing interests.

## Authors' contributions

TvG performed the assembly using CLC software, and SP performed the assembly using CAP3 software. AS and PA participated in the design and interpretation of the results and writing the manuscript. AS performed the comparison between the two assemblers and their effects on SNP markers detection. JvT and RV participated in the coordination of the study. All authors read and proved the final manuscript.
